# Supplementary material for: MetaRibo-Seq measures translation in microbiomes
Source: Nat Commun. 2020 Jun 29;11:3268. doi: 10.1038/s41467-020-17081-z (PMC7324362; doi:10.1038/s41467-020-17081-z)
Supplement: Supplementary file 10 — Supplementary Data 7 [file 41467_2020_17081_MOESM10_ESM.zip › File2/Confidence_High_Taxonomy/291593_out.krona.html]

Javascript must be enabled to view this page.

members
magnitude
magnitudeUnassigned
count
unassigned
taxon
rank

291593\_out

23

2
23
superkingdom

976
23
phylum

class
23
200643

171549
23
order

family
171550
23

239759
23
genus

species

SRS012969\_contig\_number\_13711SRS014613\_contig\_number\_19249SRS017103\_contig\_number\_21706SRS017916\_contig\_number\_9457SRS019496\_contig\_number\_17202SRS045826\_contig\_number\_626SRS046717\_contig\_number\_9578SRS053649\_contig\_number\_17135SRS063190\_contig\_number\_contig-100\_1176.73776SRS064276\_contig\_number\_24382SRS075821\_contig\_number\_22290SRS076756\_contig\_number\_1484SRS077194\_contig\_number\_29419SRS097958\_contig\_number\_5778SRS098571\_contig\_number\_23472SRS098644\_contig\_number\_17702SRS098717\_contig\_number\_3322SRS1041031\_contig\_number\_23428SRS104693\_contig\_number\_10954SRS144297\_contig\_number\_10512SRS144506\_contig\_number\_30495SRS148424\_contig\_number\_3306SRS893373\_contig\_number\_22342
23
1118061
